# Effects of rosiglitazone on contralateral iliac artery after vascular injury in hypercholesterolemic rabbits

**DOI:** 10.1186/1477-9560-6-4

**Published:** 2008-05-16

**Authors:** Olímpio Ribeiro França Neto, Dalton Bertolim Precoma, Alexandre Alessi, Camila Prim, Ruy Fernando Kuenzer Caetano da Silva, Lucia de Noronha, Liz Andréa Villela Baroncini

**Affiliations:** 1 Center of Health and Biological Sciences-Pontifical Catholic University of Paraná, Rua Imaculada Conceição, 1155, Prado Velho, CEP: 80215-901 Curitiba- Paraná –, Brazil

## Abstract

**Background:**

The objective was to evaluate the effects of rosiglitazone on iliac arteries of hypercholesterolemic rabbits undergoing balloon catheter injury in the contralateral iliac arteries.

**Methods:**

White male rabbits were fed a hypercholesterolemic diet for 6 weeks and divided into two groups as follows: rosiglitazone group, 14 rabbits treated with rosiglitazone (3 mg/Kg body weight/day) during 6 weeks; and control group, 18 rabbits without rosiglitazone treatment. All animals underwent balloon catheter injury of the right iliac artery on the fourteenth day of the experiment.

**Results:**

There was no significant difference in intima/media layer area ratio between the control group and the rosiglitazone group. Rosiglitazone did not reduce the probability of lesions types I, II, or III (72.73% vs. 92.31%; *p *= 0.30) and types IV or V (27.27% vs. 7.69%; *p *= 0.30). There were no differences in the extent of collagen type I and III deposition or in the percentage of animals with macrophages in the intima layer. The percentage of rabbits with smooth muscle cells in the intima layer was higher in rosiglitazone group (*p *= 0.011).

**Conclusion:**

These findings demonstrate that rosiglitazone given for 6 weeks did not prevent atherogenesis at a vessel distant from the injury site.

## Background

Balloon angioplasty is a common intervention for treatment of blood vessel stenosis, particularly in coronary vasculature. However, the balloon directly inflicts significant trauma to the vascular endothelium, which is evidenced by an immediate loss of endothelial-dependent relaxation, and is associated with a concomitant induction of a smooth muscle cell (SMC) proliferation and neointimal formation [1]. There is some evidence that balloon injury can induce physiological changes at sites anatomically distant from, as well as at the site of the injury itself [1,2]. Accorsi et al [2] presented potential systemic adverse effects of angioplasty in a rat injury model. In this experiment the authors showed that following balloon injury, there was a delayed hyper-reactivity to both phenylephrine and angiotensin II in contralateral rat carotid arteries, occurring between 4–7 and 15–30 days, respectively, and thereafter returning to control levels. Peroxisome proliferator-activated receptor γ (PPAR-γ) is a member of the nuclear receptor superfamily, which when activated by thiazolidinedione (TZD) insulin sensitizers, regulates the expression of genes that control lipid and glucose homeostasis, thus modulating the major metabolic disorders predisposing to atherosclerosis [3-5]. All of the major cells in the vasculature express PPAR-γ, including endothelial cells, vascular smooth muscle cells (VSMC), and monocytes/macrophages [3,4]. Rosiglitazone, a PPAR-γ agonist, can prevent neointimal formation and reduce macrophage content in the carotid artery of a mouse injury model of type 2 diabetes [6]. In a combined diabetes-atherosclerosis mouse model, Levi et al [7] suggested a direct anti-atherogenic effect of rosiglitazone on the arterial wall, despite higher lipid levels and similar glucose levels. The use of PPAR-γ agonist rosiglitazone (RZG) for 5 weeks in hypercholesterolemic rabbits exert significant endothelial protection by antioxidative and antinitrative effects [8]. The purpose of the present study was to investigate the effects of rosiglitazone on the contralateral unballooned iliac artery in hypercholesterolemic rabbits.

## Methods

Thirty-two white adult male rabbits (New Zealand), weighing 2.596 ± 530 Kg, were used for this experiment. Animals were handled in compliance with the Guiding Principles in the Care and Use of Animals. Protocol approval was obtained from the Pontifical Catholic University Animal Research Committee. During first 14 days, the animals were fed a hypercholesterolemic diet (1% cholesterol-Sigma-Aldrich^®^). Subsequently, they were fed a 0.5% cholesterol diet until sacrifice (45 days). The animals were divided into two groups as follows: control group (CG), 18 rabbits without RGZ; and rosiglitazone group (RG), 14 rabbits treated with RGZ administered by oral gavage (3 mg/Kg body weigh/day) during all the experiment.

### Vascular injury

All the animals underwent balloon catheter (20 × 3 mm/5 atm/5 min) injury of the right iliac artery on the fourteenth day of the experiment. Anesthesia was induced with ketamine (Vetanarcol^®^-König – 3.5 mg/Kg) and intramuscular xylazine (Coopazine^®^- Coopers – 5 mg/Kg). After the procedure animals had received intramuscular analgesics for 3 days (25 mg/day of flunixin – Banamine^® ^-Schering-Plough) and intramuscular antibiotics for 4 days (100 mg/day of oxitetracyclin – TormicinaP^®^- Toruga). Rabbits were sacrificed by a lethal barbiturate dose on day 45 and their unballooned contralateral iliac arteries were removed for immunohistochemical and histological analysis.

### Quantitative histopathology

Histological analysis was done by an experienced pathologist (LN) who was unaware of the RGZ treatment, with a microscope attached to the Image Pro-plus^® ^4.5 Software (Media Cybernetics Inc. Siver Spring, MD. USA). Histomorphometric parameters were performed with calculation of the intima/media layer area ratio (the area of the intima layer divided by the area of the media layer) according to method described by Phillips et al [5]. The quantification of type I and type III collagen area was made by the Sirius red polarization method [9]. Atherosclerotic lesions were analyzed and classified according to Stary et al [10-12].

### Immunohistochemistry

Tissue preparation and immunohistological techniques were performed according to the manufacturer's instructions included in the kits (Dako Corporation, Carpinteria, Calif). Sections were stained for macrophage cells using primary monoclonal antibody RAM-11 (Dako^®^, Carpinteria, CA), and for alpha-actin smooth muscle cells with primary polyclonal antibody HHF-35 (Dako^®^, Carpinteria, CA). For qualitative immunohistochemical comparisons of macrophage and smooth muscle cell presence in the intima area, sections were computed and scored in percentages of animals with cells in the unballooned iliac artery. For quantitative immunohistochemical comparisons of macrophage or smooth muscle cell content in intima area, sections were computed and scored in percentages of cells in the intima.

### Blood chemistry

Blood samples were obtained on first day experiment, immediately before balloon catheter injury, and immediately before sacrifice by cardiac puncture. Clinical laboratory assessment included fasting serum glucose, total cholesterol (TC), high-density lipoprotein cholesterol (HDL-C), and triglycerides (TGC). Measurements were done using an automated system (Abbott Architect ci8200; Abbott Laboratories, Abbott Park, III).

### Statistical analysis

The calculation of sample size was done based on the study of Wang Zhao-hui, Luo Feng and Liu Xiao-mei [13]. The main variable of interest was considered to be the ratio between the intima layer and the media layer. To detect a minimum difference of 0.15 between the averages of groups, with a significance level of 5% and power of the test by 80%, the minimum number of animals in each group of the study was defined as 12. Categorical variables were expressed as percentages and continuous variables were expressed as mean ± SD and medians. Shapiro-Wilks test was used for testing sample normality. For quantitative parameters, the Student *t*-test and Mann-Whitney nonparametric test were used for the comparison between CG and RG. Fisher's exact test was used for qualitative or categorical variables. Statistical significance was indicated by a value of *p *< 0.05. Analyses were performed using Statistica/W version 5.1 (StatSoft, Tulsa, Okla.).

## Results

### Metabolic and lipid profiles

Rabbit's weight did not differ between groups (data not shown). Baseline glucose, total cholesterol, HDL- cholesterol and triglycerides levels were equal in both groups before initiation of the diet. A graded elevation in TC and glucose levels was observed from the initial phase through the sacrifice in both groups. At the time of euthanasia the glucose levels did not differ between groups. Higher levels of TGC and HDL-C and lower levels of TC were observed in RG versus CG at the time of sacrifice (Table 1).

**Table 1:** Metabolic and lipid profiles (mean ± sd).

		CG	RG	*P *value
Baseline	TC (mg/dl)	39.67 ± 12.76	38.93 ± 10.92	0.837
	*HDL-C (mg/dl)	*25 (10 – 44)	*19 (12 – 36)	0.156
	TGC (mg/dl)	85.28 ± 51,60	81.36 ± 33,44	0.807
	Glucose (mg/dl)	121.89 ± 15.40	117.36 ± 11,19	0.362
Sacrifice	TC (mg/dl)	864.22 ± 269.85	638.29 ± 269.54	0.026
	*HDL-C (mg/dl)	*28 (13 – 125)	*54 (27 – 85)	0.020
	TGC (mg/dl)	117.61 ± 75.07	192.50 ± 87.21	0.01
	Glucose (mg/dl)	195.83 ± 70.15	237.86 ± 87.43	0.142

(*) HDL-C values are expressed as median, maximum and minimum.

### Histomorphometry

**Figure 1:**
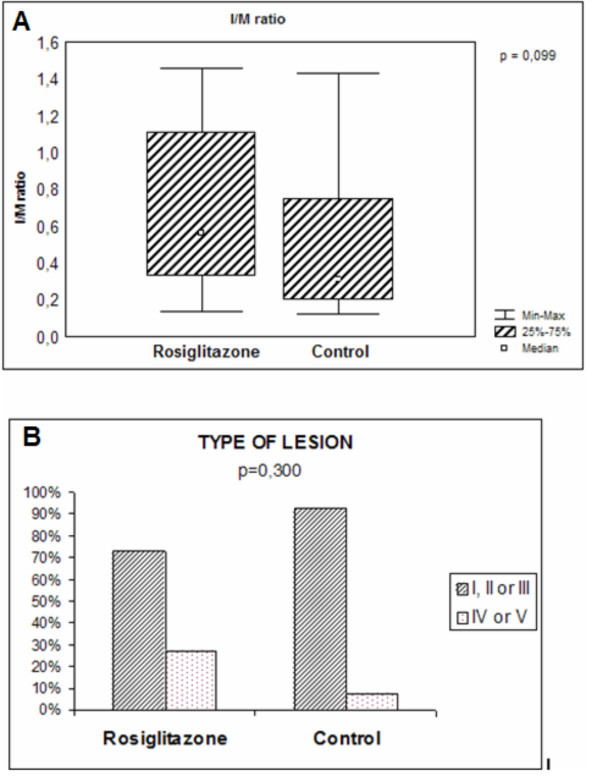
Graphics of intima/media layer ratio and histologic classification of lesions. **A**. Intima/media layer area ratio. **B**. Incidence of lesions types according with histological classification.

There was no significant difference in intima/media layer area ratio between CG and RG (Figure 1). According to histological classification proposed by Stary et al, rosiglitazone did not reduce the probability of lesions types I, II, or III (72.73% vs. 92.31%; *p *= 0.30) and types IV or V (27.27% vs. 7.69%; *p *= 0.30) when compared to CG (Figure 1). In addition, there were no differences in the extent of collagen type I and III deposition between CG and RG (data not shown).

### Immunohistochemistry

**Figure 2:**
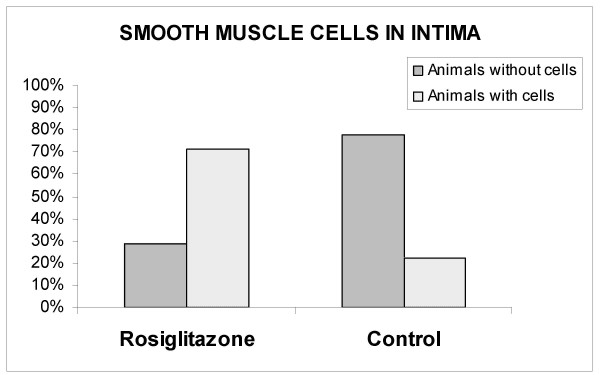
Percentage of animals with and without smooth muscle cells in the intima layer.

**Figure 3:**
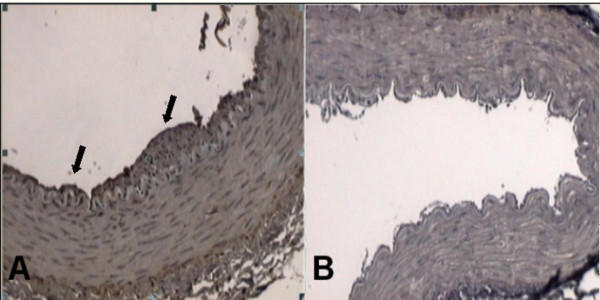
Smooth muscle cells presence. **Panel A**. Smooth muscle cells stained with HHF 35 in the intima layer (black arrows) of a rabbit from Rosiglitazone group. **Panel B**. Absence of smooth muscle cells stained with HHF35 in the intima layer of a rabbit from Control group.

There was no statistically significant difference in the percentage of animals with macrophages in the intima layer between CG and RG (33.4% vs. 71.5%; *p *= 0.07). The percentage of animals with smooth muscle cells in intima layer was higher in RG when compared with CG (22.3% vs. 71.5%; *p *= 0.011). (Table 2). (Figures 2 and 3).

**Table 2:** Percentage of macrophage and SMC in intima layer.

	CG (N/%)	RG (N/%)
Animals with macrophage (*p *= 0.07)	6 (33.4%)	10 (71.5%)
≤ 10% cells	3 (16.6%)	5 (35.7%)
10–25% cells	1 (5.5%)	0
25–50% cells	2 (11.1%)	5 (35.7%)
50–75% cells	0	0
> 75% cells	0	0
Animals with SMC (*p *= 0,01)	4 (22.3%)	10 (71.5%)
≤ 10% cells	1 (5.5%)	3 (21.4%)
10–25% cells	0	0
25–50% cells	2 (11.1%)	5 (35.7%)
50–75% cells	1 (5.5%)	2 (14.2%)
> 75% cells	0	0

## Discussion

In this study, we analyze the histological changes caused by catheter balloon injury and the potential effects of rosiglitazone on a vessel distant from the injury site. We used rabbits with six-fold increased cholesterol levels at the time of vascular injury and fourteen-fold increased levels at the time of euthanasia. Previous studies have shown that PPARγ is highly expressed in macrophage foam cells from atherosclerotic lesions and has been demonstrated in cultured macrophages to both positively and negatively regulate genes implicated in the development of atherosclerosis [14,15]. Collins et al [16] demonstrated that PPARγ troglitazone inhibited lesion formation in male low density lipoprotein receptor-deficient mice fed with either a high-fat diet or a high-fructose diet by decreasing the accumulation of macrophages in intimal xanthomas. In contrast, Hennuyer et al [5] reported that in a dyslipidemic nondiabetic murine model (E2-KI mice), PPARα, but not PPARγ (rosiglitazone and pioglitazone), activators protect against macrophage foam cell formation. This can be explained by the fact that in the presence of severe dyslipidemia PPARγ activation in macrophages is insufficient to reverse the pro-atherogenic process. Chawla et al [17], using LDLR -/- mice, showed that in addition to lipid uptake, PPARγ regulates a pathway of cholesterol efflux. Another possible explanation for these findings, raised by the authors, is that homeostasis cholesterol control by activation of PPARγ may differ between species [15,18]. Wang et al [13] showed a reduction in aorta intima/media ratio in hypercholesterolemic rabbits receiving RGZ (0.5 mg/weight) for 6 weeks. Our data showed no significant difference regarding the percentage of animals with intimal macrophages, initial and advanced atherosclerotic lesions, and intima/media layer ratio in contralateral iliac artery with the use of RGZ. This can be explained, in part, by the fact that PPARγ agonist stimulate expression of the scavenger receptor CD36 in macrophages that facilitates uptake of oxidized LDL and contributes to the development of atherosclerosis [18-20]. Li et al [14,15] reported that rosiglitazone and GW7845 strongly inhibited the development of atherosclerosis in LDL receptor-deficient male, despite their proatherogenic effects evidenced by increased expression of the CD36 scavenger receptor in arterial wall. In the present study, we did not evaluate the expression of CD36. It has also been suggested that the effects of RGZ in foam cell formation and atherosclerosis may differ in the degree of insulin resistance [5,21]. We found a graded and significant elevation of glucose levels from the initial phase through the sacrifice in both groups with and without RGZ and we believe that it could be secondary to the development of some degree of insulin resistance although this was not evaluated in the present study. Wang et al [13] did not show a reduction of blood glucose level in hypercholesterolemic rabbits receiving RGZ for 6 weeks, as in the present study. Furthermore, we also observed a significant elevation of triglycerides and HDL-C at the time of euthanasia in RGZ group. The effects of thiazolidinediones on triglycerides have been somewhat more variable. Decreases in triglyceride levels have been more frequently observed with pioglitazone than with rosiglitazone. We cannot rule out that these effects on glucose and triglycerides were due to chance, as our evaluation period was short and the sample was relatively small. These findings are quite controversial in the literature [7,8,13]. Other studies with different animals-models [1,2,22] have related a potential increase in cell replication far from the injury site and endothelial dysfunction in contralateral artery after balloon catheter injury. We have found a significant increase of animals with SMC in the intima layer of unballooned iliac arteries. While in other studies there is evidence of antiatherogenic effects of these drugs in different animal models and in diabetic patients [23-28], this is the first study to report a lack of antiatherogenic effects of a PPARγ agonist in a vessel distant from the injury site. We cannot rule out that our histological analysis reflected a short period of exposure to RGZ. In addition, we did not evaluated the artery vasodilatation, peroxynitrite (ONOO^-^) formation, endothelial nitric oxide (NO), or the expression of vasodilator-stimulated phosphoprotein VASP (P-VASP) that should be assessed in future studies. The presence of diabetes accelerates the process of atherosclerosis and cardiovascular disease is the leading cause of death and the major cause of morbidity [29-31]. Thus, it is necessary to enhance our knowledge about the mechanisms of action of thiazolidinediones largely used in type 2 diabetes, particularly rosiglitazone that has been the focus of extensive discussion in recent publications. Nissen and Wolski [29] published a meta-analysis showing a significant increase in the risk of myocardial infarction and an increase in cardiovascular death of borderline significance in patients with diabetes receiving RGZ. Singh et al [32] also published a meta-analysis showing a significantly increased risk of myocardial infarction and heart failure among patients with impaired glucose tolerance or type 2 diabetes using rosiglitazone for at least 12 months, with no significantly increased risk of cardiovascular mortality. Lipscombe et al [33], in a nested case-control analysis of a retrospective cohort study, found that in diabetes patients with an age of 66 years or older, RGZ treatment was associated with an increased risk of congestive heart failure, acute myocardial infarction, and mortality when compared with other combination oral hypoglycemic agent treatments. The mechanism for the apparent increase in myocardial infarction and death from cardiovascular causes associated with RGZ remains uncertain. However, to apply the effects of thiazolidinediones on atherosclerosis in experimental models to the clinical practic is difficult and largely depends on the validity of the model. In the next three years, we hope that the final results of the studies RECORD and BARI-2D [30,34], specifically evaluating cardiovascular effects of RGZ, will provide useful insights.

## Competing interests

The authors declare that they have no competing interests.

## Authors' contributions

ORFN, DBP, and AA had designed the study.

CP oriented in the management of the animals.

RFKCS oriented in the surgical procedures.

LN made the histological examination of the iliac arteries

LAVB wrote and oriented the manuscript.

All authors read and approved the final manuscript.
